# Through the Foramen and Beyond: A Case Report of Internal Herniation of Caecum Through the Epiploic Foramen of Winslow

**DOI:** 10.7759/cureus.97391

**Published:** 2025-11-20

**Authors:** Kyle Walsh, Anna Murray, Claire Jones, Andrew McGuigan

**Affiliations:** 1 General Surgery, Southern Health and Social Care Trust, Craigavon, GBR; 2 General Surgery, Southern Health and Social Care trust, Craigavon, GBR

**Keywords:** blandin's hernia, epiploic foramen, foramen of winslow, internal hernia, lesser sac, lesser sac hernia, strangulated internal hernia, surgical case reports

## Abstract

Internal herniae are a rare subtype of hernia defined by the protrusion of a viscus through the opening in the peritoneum or mesentery within the abdominal cavity. Herniation into the lesser sac through the epiploic foramen of Winslow is an uncommon subclassification of internal abdominal hernia. They are an important clinical entity and carry a high mortality risk if strangulation is present. This case report describes an 82-year-old female patient who presented with an acute onset of abdominal pain and distension. Initial computerised tomography imaging suggested caecal volvulus as a working diagnosis, prompting an emergency laparotomy. Intraoperatively, a right hemicolectomy was performed for a strangulated caecum herniated into the lesser sac via the epiploic foramen of Winslow. Postoperatively, this patient recovered well. This rare subtype of internal hernia can be challenging to diagnose preoperatively, leading to a delay in surgical intervention, conveying a high mortality rate. Our case highlights that early intervention in this setting leads to good post-operative outcomes.

## Introduction

An internal hernia is defined as the protrusion of a viscus through an opening in the peritoneum or mesentery within the abdominal cavity [1]. Despite being relatively rare (1% of all hernias), they are often problematic, with 0.6-5.8% of all small bowel obstructions having an internal hernia as the underlying cause [2]. Internal hernias can be congenital or acquired following operative intervention or intra-abdominal inflammation.

An epiploic hernia, eponymously known as a Blandin's hernia, is a rare type of abdominal hernia with an incidence of <0.1% [3]. Most commonly, the small bowel is implicated in any internal herniae, and the same applies to herniae through the epiploic foramen [4]. These are an important clinical entity that carry a 50% risk of mortality if strangulation is present [2]. Rarely, the caecum can herniate through the epiploic foramen of Winslow with a reported incidence of 0.02% [5]. Very few case reports exist depicting a strangulated caecal volvulus in the epiploic foramen of Winslow [5]. This case report adds to the scarce literature of this uncommon but important surgical presentation. This article presents the case of an 82-year-old female patient who presented to the emergency department symptomatic of the internal herniation of a strangulated caecum into the lesser sac via the epiploic foramen of Winslow. Covered within this report are the presentation, investigations, treatment, and outcome, as well as a review of herniae through the epiploic foramen of Winslow, with a particular focus on caecal herniation.

## Case presentation

A fit and well 82-year-old female with a past medical history of hypertension presented to the emergency department (ED) with an acute onset of a 24-hour history of central abdominal pain and distension. She reported nausea and vomiting, and last had a bowel movement the day prior to attending the ED. She had no past surgical history. On examination, her observations were all within normal limits, and she had gross abdominal distension with tenderness. Differential diagnoses included small or large bowel obstruction, mesenteric ischaemia, and acute pancreatitis. Written informed consent was obtained from the patient for this case to be published, including case history, imaging, and data.

Investigations

**Figure 1:**
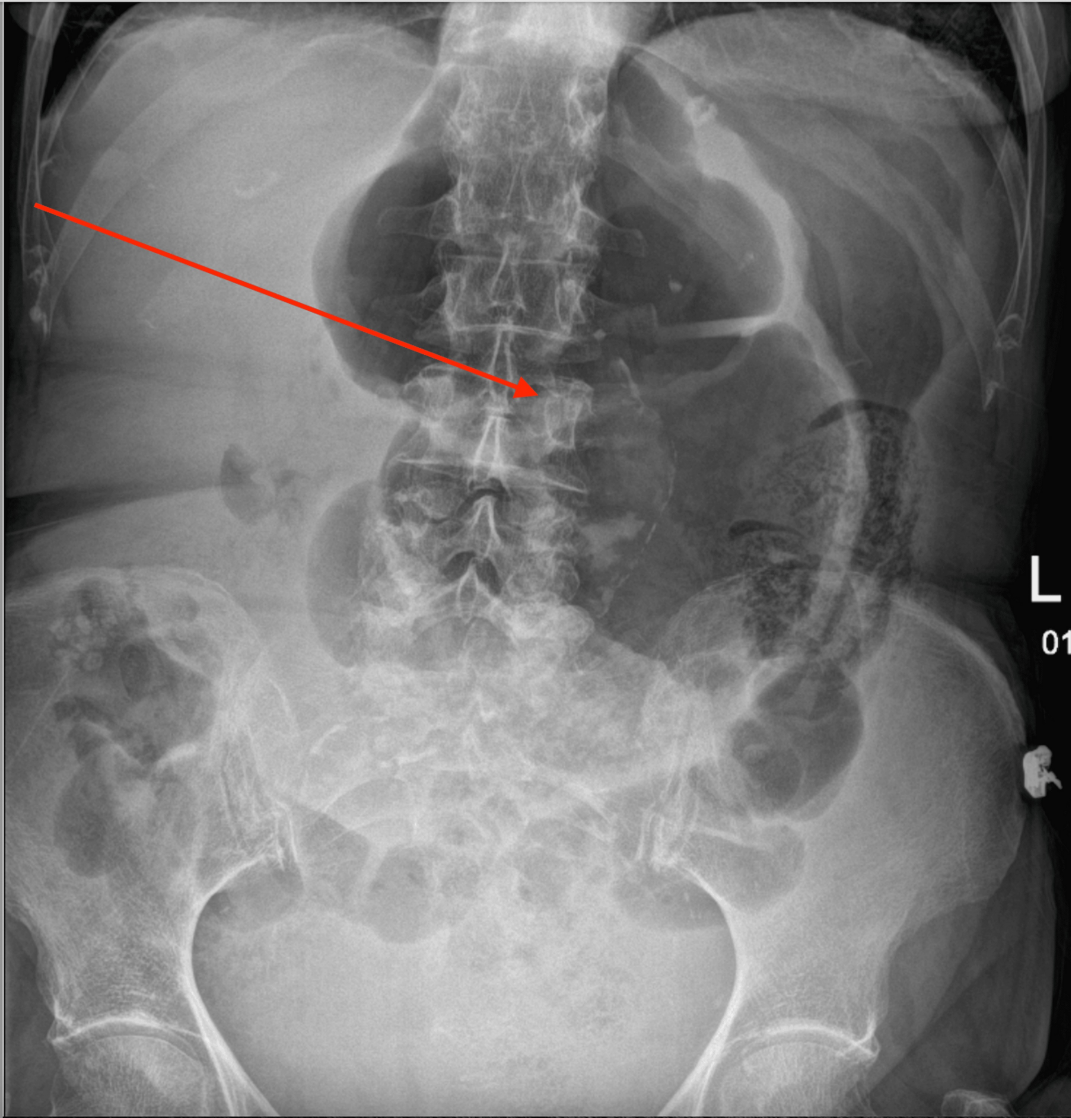
Abdominal X-ray demonstrating a centrally positioned dilated loop of colon (red arrow). Appearance similar to the 'embryo sign' seen in caecal volvulus.

**Figure 2:**
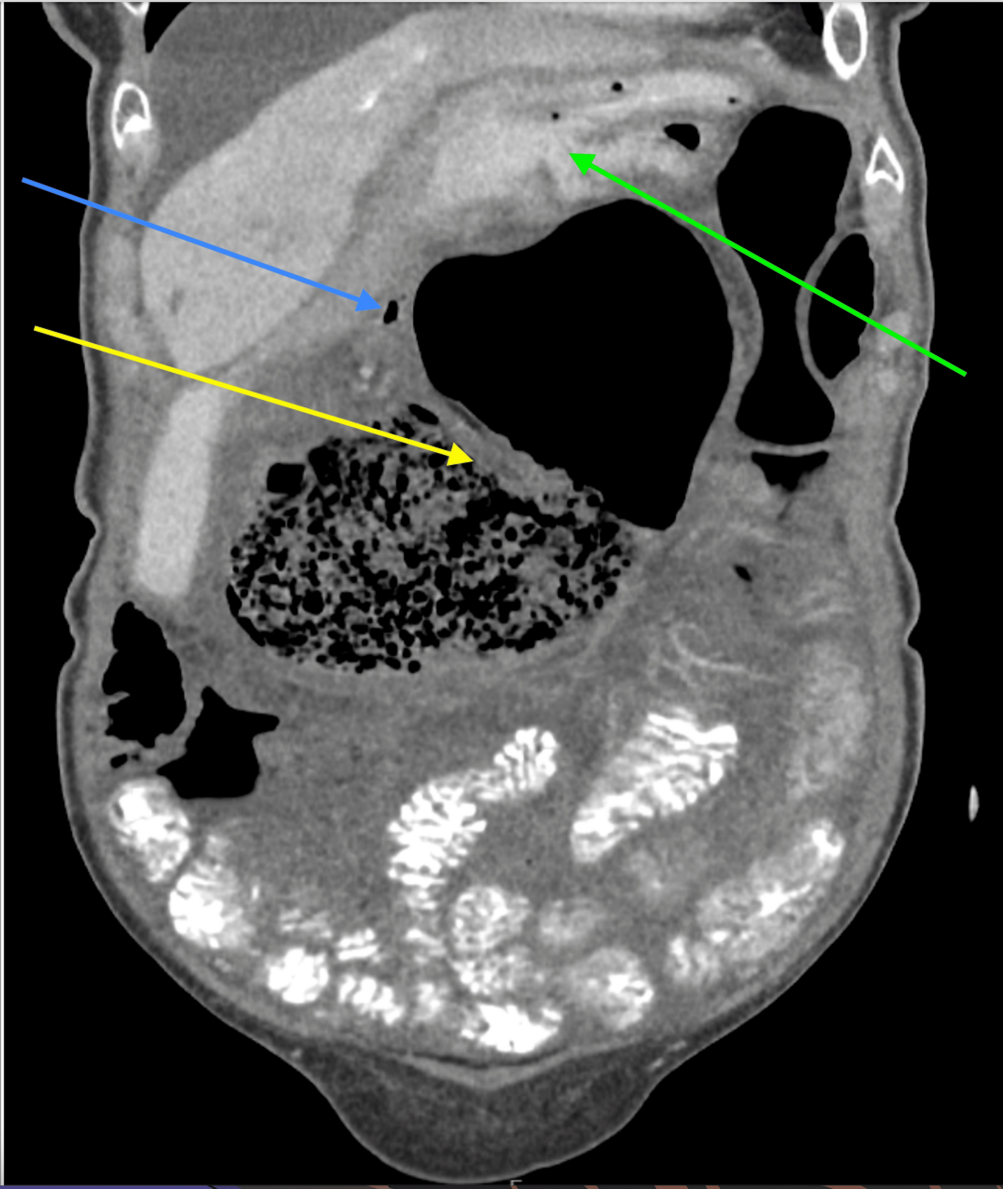
A coronal view of CT scan of the abdomen and pelvis demonstrating a malpositioned, dilated air and fluid filled caecum (yellow arrow). Closely related and identified by intra-luminal gas of the terminal ileum (blue arrow). The stomach, is displaced superiorly (green arrow).

Blood tests at presentation demonstrated a normal white cell count (9.6x10^12^/L) with a neutrophilia (8.5x10^9^/L) and C-reactive protein (0.6mg/L), hypokalaemia (3.3mmol/L) with a raised urea (8.8 mmol/L). Venous blood sampling revealed a raised lactate at 3.2mmol/L (Table 1). An erect chest radiograph demonstrated no pneumoperitoneum. A plain abdominal film (Figure 1) revealed a centrally placed loop of distended colon, akin to the typical embryo sign seen in caecal volvulus. A computed tomography (CT) scan of the abdomen and pelvis with both oral and intravenous contrast was sought. CT imaging (Figure 2) demonstrated a focally dilated loop of colon measuring up to 6cm located centrally in the abdomen. This was identified as the caecum due to its association with the terminal ileum and ileocaecal valve. This loop was located inferiorly to the stomach. The caecum was demonstrating wall hypoenhancement suggestive of colonic ischaemia. Faecalisation of the terminal ileum was present, indicating intestinal obstruction. There was associated twisting of mesenteric vessels noted adjacent to this area of concern, pre-operatively raising the possibility of a caecal volvulus.

**Table 1:** A table demonstrating the salient blood test results at presentation

Blood test	Result	Normal range
White cell count (x10^12^/L)	9.6	3.6-11
Neutrophil count (x10^9^/L)	8.5	1.8-7.5
C-reactive protein (mg/L)	0.6	<5
Potassium (mmol/L)	3.3	3.5-5.3
Urea (mmol/L)	8.8	2.5-7.8
Lactate (mmol/L	3.2	0.5-2.2

Treatment and outcome

A pre-operative working diagnosis of a caecal volvulus was made, and the patient proceeded to an emergency laparotomy after intravenous fluid resuscitation, and a nasogastric tube was placed. Intraoperatively, a dusky, grossly distended, non-viable caecum was identified posterior to the lesser omentum. The gastrocolic ligament was divided, and the lesser sac was entered. The caecum was found to have herniated via the epiploic foramen of Winslow into the lesser sac. The caecum was decompressed to facilitate reduction back through the epiploic foramen. A right hemicolectomy was then performed without primary anastomosis, and an end ileostomy was fashioned in the right iliac fossa. The foraminal defect was not closed to avoid iatrogenic injury to the portal triad. A large-bore abdominal drain was placed in the lesser sac. She was transferred to our post-anaesthetic care unit for postoperative care. This patient recovered well and was discharged home.

## Discussion

Anatomy

The epiploic foramen of Winslow was first described by Jacob Winslow in 1732. It is a naturally occurring defect in the lesser sac that allows communication between the greater and lesser sac. The foramen is bounded by the hepatoduodenal ligament (containing the portal vein, hepatic artery, and common bile duct) anteriorly, the peritoneal covering of the inferior vena cava posteriorly, the peritoneal covering of the caudate lobe of the liver superiorly, and inferiorly by the peritoneal covering of the first part of the duodenum [6]. It has relevant clinical significance as a site for internal herniation as described in this report, but additionally is commonly known as an anatomical landmark in the 'Pringle Manoeuvre'.

Incidence

Internal hernias involving the epiploic foramen constitute approximately 8% of all abdominal hernias [7]. Of all internal hernias involving the epiploic foramen, caecal involvement represents 25-30% [8]. Most commonly, the small bowel herniates through this opening, but case reports in the literature describe herniation of the gallbladder [9], omentum, and even a Meckel's Diverticulum [10]. The incidence of herniae involving the foramen of Winslow typically peaks between the ages of 20-60 years of age and demonstrates a predominance in males (male:female ratio of 2.5:1) [11].

Risk factors

Typically, this type of herniation is rare owing to the anatomy and orientation of the foramen. Predisposing factors that may increase the risk of herniae through the foramen of Winslow include a large opening of the foramen of Winslow, raised intra-abdominal pressure, as well as mobile viscera, such as that seen with long mesenteries. In this case, a highly mobile caecum is likely the cause, which arises from failure of the caecum and ascending colon to retroperitonealise embryologically. Other risk factors for a highly mobile caecum include chronic constipation, paralytic ileus, as well as neurofibromatosis [12].

Clinical relevance and diagnostic uncertainty

Although a rare entity, this is an important and clinically relevant subtype of internal hernia given its high mortality rate, reported up to 49% [13]. The high mortality rate can be attributed to strangulation and the closed-loop nature with ischaemia at presentation owing to the size of the aperture of the foramen. Additionally, as seen in our case, the high mortality rate can be attributed to the difficulty in achieving a pre-operative diagnosis, leading to a delay in intervention. Across the available literature, an accurate pre-operative diagnosis is made in less than 10% of cases [14]. In our circumstance, the CT report and discussion with radiology prompted an urgent laparotomy based on a pre-operative working diagnosis of caecal volvulus with ischaemia. In this regard, early recognition of the need for an emergency laparotomy for our patient enhanced perioperative morbidity and mortality. Early recognition and surgical intervention in this instance can reduce mortality to 5% [15].

The main learning points from this case are, firstly, herniation into the lesser sac is a rare but important cause of bowel obstruction. The management involves fluid resuscitation, placement of a nasogastric tube, and urgent surgical intervention to decompress and resect non-viable bowel. It is important to note that epiploic herniae are at high risk of strangulation and carry a high mortality rate due to difficulty in achieving an accurate preoperative diagnosis and delay in surgical intervention

## Conclusions

Internal herniae are an uncommon but important type of hernia with significant clinical relevance. Herniae of the caecum through the epiploic foramen of Winslow are a rare subtype of Blandin's hernia. They can be challenging to diagnose preoperatively, leading to a delay in surgical intervention, resulting in a high mortality rate. Our case demonstrates the difficulty in reaching an accurate pre-operative diagnosis, but also that early intervention leads to good postoperative outcomes. This case report adds to the small but growing number of case reports in the literature, raising awareness of this acute surgical presentation.
